# Synaptic mitochondrial dysfunction and Alzheimer’s disease: from molecular mechanisms to therapeutic strategies

**DOI:** 10.3389/fphar.2026.1849218

**Published:** 2026-09-03

**Authors:** Yuxin Wang, Weidong Wu, Lishuang Yan, Bo Zhang, Quan Li, Yanyan Zhou

**Affiliations:** 1 School of Basic Medicine, Heilongjiang University of Chinese Medicine, Harbin, China; 2 Key Laboratory of Basic Theory Research on Traditional Chinese Medicine, Harbin, Heilongjiang, China

**Keywords:** AD, Alzheimer’s disease, oxidative stress, ROS, synaptic mitochondrial dysfunction

## Abstract

The ability of AD treatments targeting classic pathological proteins to achieve meaningful clinical outcomes has been severely limited, shifting attention to the earlier upstream pathways that drive disease progression. Increasing evidence indicates that synaptic mitochondrial dysfunction is an early pathological event that directly contributes to synaptic loss and cognitive decline. This review focuses on how four interrelated pathologies—disrupted energy metabolism, calcium overload, imbalanced mitochondrial fission/fusion, and defective autophagy—converge to impair synaptic function and plasticity. Emerging therapeutic strategies aimed at protecting and restoring synaptic mitochondrial health, including mitochondria-targeted antioxidants, metabolic modulators, calcium signaling inhibitors, dynamics regulators, and autophagy inducers, are also examined. A central focus of the review is clinical translation: we summarize the preclinical evidence and critically evaluate major obstacles such as the lack of synapse-specific biomarkers, challenges in blood–brain barrier penetration and targeted delivery, and substantial patient heterogeneity. Rather than proposing a fully defined translational framework, we highlight the essential requirements for building one, centered on synaptic mitochondrial bioenergetics and quality control. Specifically, early and accurate biomarkers must be developed, patients should be stratified promptly, and rational combination therapies with complementary mechanisms need to be implemented. This organelle-centered perspective will clarify AD pathogenesis and help guide the development of next-generation neuroprotective therapies.

## 1 Introduction

Alzheimer’s disease (AD) is the leading neurodegenerative disorder, characterized by progressive memory loss and cognitive decline (van Olst et al., 2025). Historically, its pathology has been defined by extracellular amyloid beta (A β ) plaques and intracellular neurofibrillary tangles. However, repeated failures of therapies targeting these hallmarks in clinical trials indicate that research should shift attention to earlier and more distal cellular processes that underlie functional impairment, rather than focusing solely on A β and tau-centered interventions (Frere and Slutsky, 2018).

**FIGURE 1:**
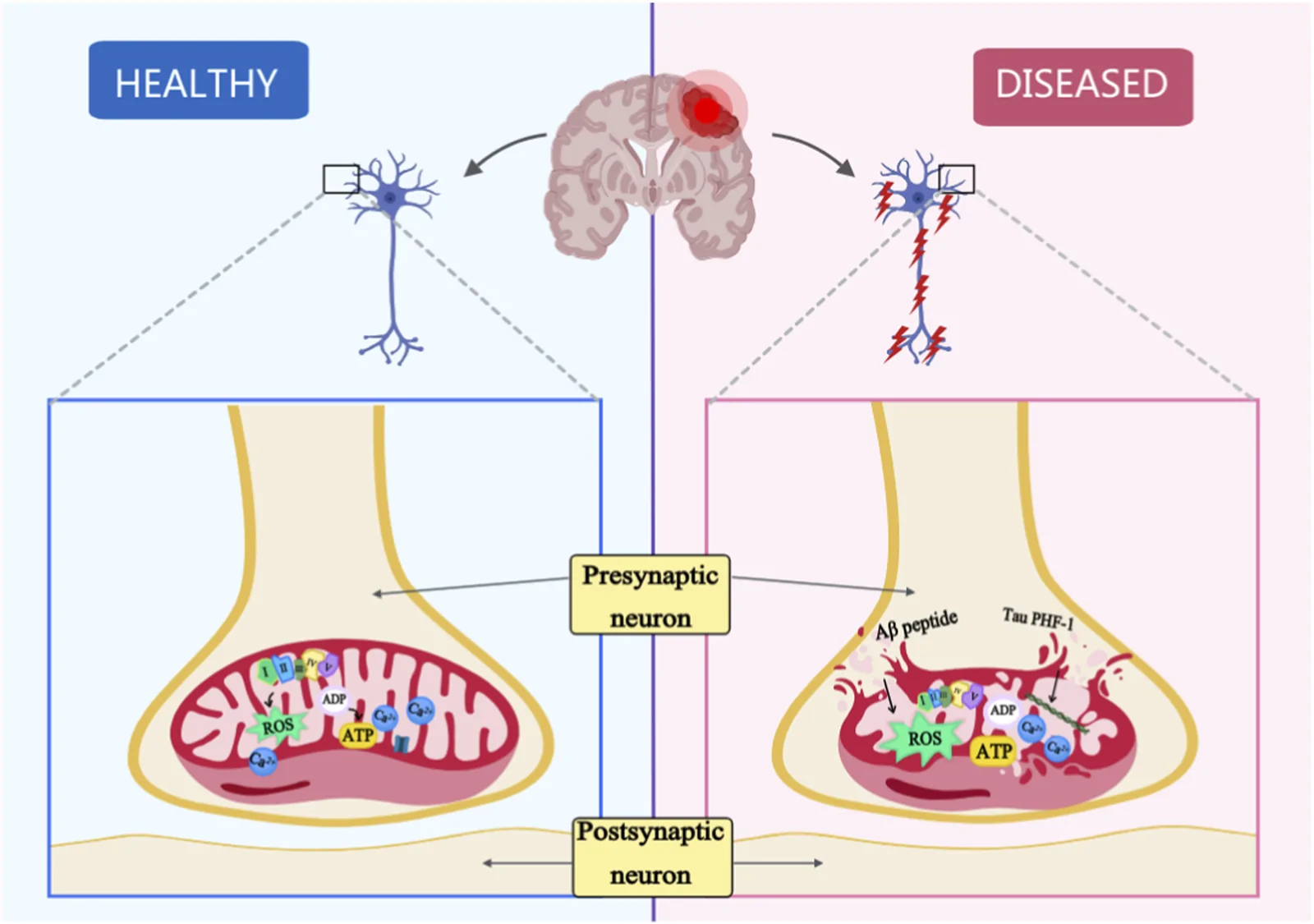
Differences in synaptic mitochondria in the brains of healthy individuals and AD patients.

Recent studies increasingly converge on early synaptic loss and dysfunction as a key pathological event: synapses are the fundamental units of neuronal information transmission and storage, and their integrity underpins cognitive function. Indeed, synaptic damage correlates more closely with cognitive decline than plaque burden (Busche et al., 2019) (Figure 1). Consequently, elucidating the initiating mechanisms of synaptic degeneration is essential both for understanding AD pathology and for developing effective therapies.

Maintaining synaptic function fundamentally requires a continuous supply of energy and intracellular homeostasis (Calvo-Rodriguez and Bacskai, 2021). Mitochondria are the principal sites of cellular ATP generation and act as signaling hubs that regulate calcium homeostasis, reactive oxygen species metabolism, and cell fate (Flippo and Strack, 2017). In highly polarized neurons, mitochondrial distribution and function are heterogeneous. Synaptic mitochondria, which are enriched at presynaptic and postsynaptic sites, display specialized and adaptive features in morphology, proteome composition, calcium-buffering capacity, and dynamic behavior. Dysfunction of these organelles is directly associated with synaptic failure (Rajendran and Paolicelli, 2018). Far from serving only as energy suppliers, mitochondria participate in nearly all core aspects of synaptic function (Smith et al., 2016).

These roles depend on precise mitochondrial transport to synaptic sites, continuous remodeling of the network through fusion and fission, and removal of damaged organelles via mitophagy (Vaccaro et al., 2017). Thus, synaptic mitochondria are essential for maintaining synaptic efficacy, plasticity, and stability.

During AD progression, this tightly regulated system sustains early and severe insult. Growing evidence shows that synaptic mitochondrial dysfunction is an early, pivotal event in AD and can precede overt A β plaque deposition and tau tangle formation (Torre et al., 2021; Reddy and Beal, 2005; de Veij Mestdagh et al., 2023). Conversely, once initiated, A β oligomers and hyperphosphorylated tau can further target and impair synaptic mitochondria, creating a self-amplifying feed-forward loop that accelerates synaptic failure. This triggers a cascade of interrelated, progressively amplifying vicious cycles: disrupted calcium homeostasis causes mitochondrial calcium overload; impaired electron transport chain function leads to ATP synthesis failure and a burst of reactive oxygen species (ROS); dysregulated mitochondrial dynamics drive excessive network fragmentation; and defective autophagic clearance permits accumulation of dysfunctional mitochondria at synapses. Together, these processes culminate in synaptic energy depletion, disrupted signal transduction, and structural collapse—in short, comprehensive synaptic failure (Zhou et al., 2021; Zhang J. et al., 2025).

Therefore, synaptic mitochondrial dysfunction constitutes a central nexus linking upstream molecular pathologies to downstream cellular phenotypes, offering a cellular framework for interventions targeting cerebral energy metabolism disturbances.

This review therefore summarizes the role of synaptic mitochondria in health and disease and examines potential therapies that target these structures. First, synaptic mitochondria are defined, and the mechanisms by which they support synaptic function are outlined. Second, the multidimensional processes that underlie synaptic mitochondrial dysfunction in AD are detailed, including loss of calcium homeostasis, a self-reinforcing cycle of impaired energy metabolism and oxidative stress, disrupted mitochondrial dynamics, and deficiencies in quality-control pathways. Third, candidate interventions directed at these nodes are assessed, such as metabolic modulation, targeted antioxidant therapies, modulation of mitochondrial dynamics, enhancement of mitophagy, and novel delivery technologies. Finally, current knowledge is summarized, and future directions are proposed, with emphasis on the notion that protecting and restoring synaptic mitochondrial function is a promising strategy to delay or halt AD progression and represents a central target for next-generation disease-modifying therapies aimed at neuronal energy metabolism.

## 2 Structural and functional characteristics and roles of synaptic mitochondria

### 2.1 Differential characteristics of synaptic versus non-synaptic mitochondria

Synaptic and non-synaptic mitochondria differ markedly in morphology, molecular composition, and function, and these differences underpin their specialized roles within neurons (Misgeld and Schwarz, 2017). Although the precise molecular mechanisms that produce these distinctions remain incompletely understood, multiple lines of investigation have characterized key differences. Morphological studies of rodent and human brain tissue show that mitochondria in presynaptic and postsynaptic compartments are generally smaller and more spherical than non-synaptic mitochondria found in neuronal somata or along axonal shafts; by contrast, non-synaptic mitochondria commonly form extended tubular networks (Graham et al., 2017; Seager et al., 2020).

The compact, spherical shape of synaptic mitochondria is thought to enhance surface-area-to-volume ratios, which would allow faster responses to local Ca^2+^ transients generated by synaptic activity and more effective buffering of those transients (Cserép et al., 2020; Fedorovich et al., 2017). Hence, this morphology may contribute to refining synaptic transmission and plasticity. However, direct experimental evidence causally linking these specific morphological properties to functional outcomes at the synapse remains limited (Liang, 2021). Mitochondria take up calcium via the mitochondrial calcium uniporter (MCU) and are crucial for maintaining neuronal calcium homeostasis. High-frequency neuronal firing induces rapid and transient increases in presynaptic Ca^2+^ levels. This heightened calcium sensitivity enhances the ability of synaptic mitochondria to buffer Ca^2+^, support neurotransmitter release, and fulfill other functions. However, it also renders them susceptible to pathological stress, including opening of the permeability transition pore, excessive production of ROS, and subsequent damage (Roman et al., 2024; Silva-Rodrigues et al., 2020; Olesen et al., 2020).

In contrast, under pathological conditions, excessive Drp1-mediated fission generates abnormally small mitochondria that, despite an apparent increase in surface area, exhibit disrupted cristae architecture and profound loss of mitochondrial membrane potential (ΔΨm). These ultrastructural defects severely impair the electrochemical driving force required for Ca^2+^ uptake via the MCU, thereby markedly compromising mitochondrial calcium buffering capacity. Moreover, the loss of membrane potential exacerbates ROS leakage and increases the propensity for permeability transition pore opening, collectively rendering synaptic mitochondria more vulnerable to degeneration.

Proteomic studies further show substantial differences in protein composition between synaptic and non-synaptic mitochondria, notably in proteins related to energy metabolism, calcium handling, antioxidant defense, and dynamics (Graham et al., 2017; Völgyi et al., 2015; Fecher et al., 2019). This specialized proteome likely accounts for their distinct functional properties and differential vulnerability to injury.

### 2.2 Transport and positioning mechanisms of synaptic mitochondria

Mitochondrial distribution in neurons is dynamic, ensuring that energy supply and calcium buffering are delivered to high-demand synaptic sites. Mitochondria are synthesized in the cell body and transported along the microtubule cytoskeleton to distal nerve terminals by active transport (Li and Sheng, 2022). During synaptic activity, mitochondria are recruited to and anchored at active pre- and postsynaptic regions (Li et al., 2020). Beyond long-range transport from the soma, local mitochondrial DNA replication and biogenesis at peripheral axonal or dendritic sites supply rapid, on-site replenishment of functional mitochondria to the synapse (Bao et al., 2023). Bidirectional axonal transport of mitochondria is essential to maintain their proper distribution and function. Anterograde transport, from the soma to distal processes, is mediated by kinesin-family motor proteins, whereas retrograde transport, from distal processes back to the soma, is driven by the cytoplasmic dynein/dynactin complex (Fenton et al., 2021).

The direction, initiation, and termination of transport are determined by adaptor complexes on the mitochondrial outer membrane. Key adaptor proteins are the calcium-sensing mitochondrial Rho GTPase 1/2 (Miro1/2) and the trafficking kinesin protein1/2 (TRAK1/2) adaptors. TRAK proteins bind simultaneously to Miro and to motor proteins, thereby loading mitochondria onto appropriate transport tracks (López-Doménech et al., 2018; van Spronsen et al., 2013).

Microtubule polarity differs fundamentally between axons and dendrites: axonal microtubules are uniformly oriented with their plus ends directed toward the periphery, so kinesin and dynein mediate largely unidirectional anterograde and retrograde transport, respectively. In contrast, dendritic microtubules exhibit mixed polarity, producing more complex mitochondrial trafficking that depends heavily on dynein-mediated movement (Kapitein and Hoogenraad, 2015). A balance between anterograde and retrograde transport is therefore crucial both to supply synapses with healthy mitochondria and to remove damaged organelles.

Multiple studies using neurodegenerative disease models show that mitochondrial trafficking is important. For example, in neurons from AD models, the oligomeric amyloid inhibits anterograde axonal transport of mitochondria (Wang N. et al., 2025), and conditional knockdown of Miro1 in mice leads to mitochondrial depletion in distal hippocampal dendrites, dendritic degeneration, and neuronal death. The Miro1-dependent transport is important for distal dendritic structure and function (López-Doménech et al., 2021).

### 2.3 Role of presynaptic mitochondria

Mitochondria at presynaptic terminals support multiple stages of neurotransmitter release by supplying ATP and buffering Ca^2+^. Experimental inhibition of presynaptic oxidative phosphorylation, which depletes ATP, substantially reduces the rate of synaptic vesicle recycling (Li et al., 2016).

In addition, the volume of the mitochondrial matrix in the presynaptic terminal correlates with the level of exocytosis. This suggests that local vesicle release depends on ATP production (Chemla et al., 2025). In addition, mitochondrial Ca^2+^ buffering regulates presynaptic Ca^2+^ transitions and neurotransmitter release probability (Ivannikov et al., 2013). Synapses without functional mitochondria release more vesicles and higher cytoplasmic Ca^2+^ when depolarized. This implies that presynaptic mitochondria are a brake against excessive neurotransmitter release and excitotoxicity (Billups and Forsythe, 2002). Blocking mitochondrial transport to presynaptic sites alters the asynchronous release pattern generated by depolarization, which suggests that local mitochondria are required to maintain normal release dynamics (Sun et al., 2013).

Mitochondrial fusion and fission strongly influence presynaptic function. Fused larger mitochondria typically have higher Ca^2+^ uptake capacity, while fragmented mitochondria with excessive fission have less Ca^2+^ buffering (Flippo et al., 2020). However, sustained, unregulated fission is also harmful. For example, presynaptic knockout of Drp1 decreases the volume of the synaptic vesicle recycling pool and vesicle recirculation in mice. This defect is partially compensated by strengthening intracellular Ca^2+^ buffering, but not by the acute addition of exogenous ATP. Presynaptic fission is more likely to affect local Ca^2+^ handling than short-term energy supply (Rodriguez Salazar et al., 2025).

### 2.4 The role of postsynaptic mitochondria

Postsynaptic mitochondria in dendritic spines and adjacent trunks are essential for spine structure and function (Faitg et al., 2021). Disrupting the function or transport of these dendritic mitochondria reduces spine density and causes synaptic loss. Genetic manipulation of mitochondrial fission is commonly used to probe postsynaptic roles: expression of a dominant-negative Drp1 in hippocampal neurons inhibits fission, lowering dendritic mitochondrial density and reducing functional postsynaptic sites (Li et al., 2004).

Conversely, Drp1 overexpression, which promotes fission, increases dendritic mitochondrial density and the number of functional synapses. Overexpression of Optic atrophy 1 (OPA1), which promotes mitochondrial fusion, did not significantly change mitochondrial number but did reduce the average length of dendritic mitochondria (Molnár, 2011). The activity of postsynaptic mitochondria is itself regulated by synaptic activity: in cultured hippocampal neurons, glutamate-induced depolarization and Ca^2+^ influx rapidly trigger mitochondrial fission and raise intramitochondrial Ca^2+^ concentration (Cheng et al., 2020).

**FIGURE 2:**
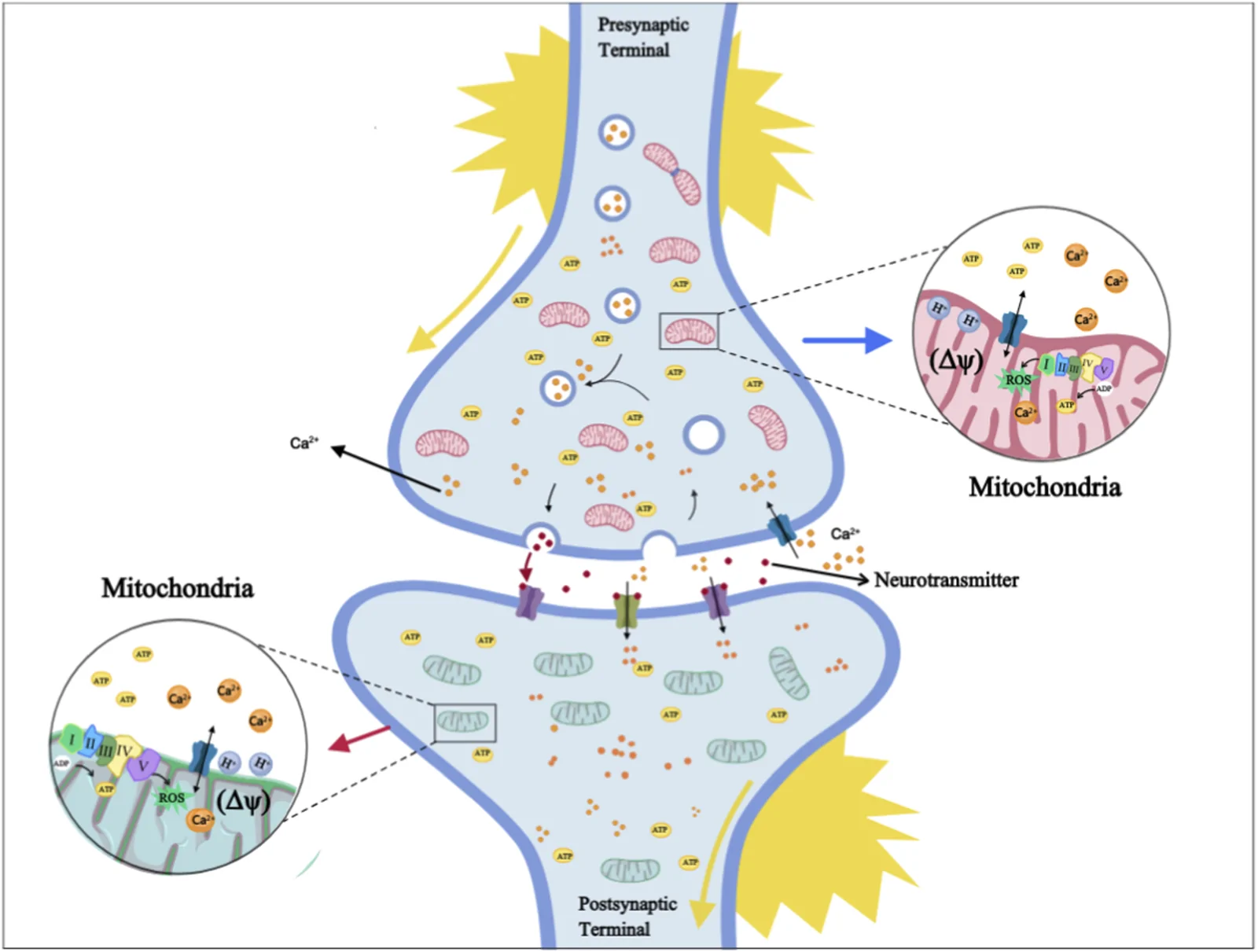
Synaptic mitochondria are essential components of both presynaptic and postsynaptic terminals. The main panel illustrates their localization and functions at the synapse. In the presynaptic terminal, mitochondria supply ATP required for synaptic vesicle cycling and the maintenance of ion gradients. In the postsynaptic compartment, mitochondria support receptor trafficking, local protein synthesis, and calcium signaling. Ca^2+^ signals are uniformly represented in orange throughout the figure. The left zoomed panel shows postsynaptic mitochondria (red arrowheads), whereas the right zoomed panel shows presynaptic mitochondria (blue arrowheads). The inset provides a close-up of the inner mitochondrial membrane, where the electron transport chain (complexes I–V) drives ATP synthesis and modulates ROS production. This integrated view illustrates how mitochondrial positioning and morphology are tailored to meet the distinct metabolic and signaling demands of pre- and postsynaptic terminals.

Another study showed that inducing fission of dendritic mitochondria to reduce their volume led to decreased frequency of postsynaptic Ca2+ transients during synaptic activity, thereby inhibiting local protein synthesis in stimulated dendritic spines (Cammalleri et al., 2003). Together, these findings indicate that postsynaptic mitochondrial fission couples synaptic activity, Ca2+ signalling, and local protein synthesis, revealing a fundamental role for mitochondrial dynamics in synaptic plasticity. At the postsynaptic terminal, mitochondria supply the ATP required for signal transduction and synaptic plasticity. Together with presynaptic mitochondria, their coordinated functions sustain the efficiency and adaptability of synaptic transmission (Figure 2).

## 3 Core mechanisms of synaptic mitochondrial dysfunction and their role in Alzheimer’s disease

Synaptic mitochondrial dysfunction follows a clear pathological cascade. Initiating insults—for example, chronic excitotoxicity, A β oligomers, or, in the subset of AD cases with concomitant Lewy body co-pathology, pathological α-synuclein—directly injure mitochondria by causing calcium overload and impairing the electron transport chain, precipitating an ATP crisis and generating ROS (Offerdahl and Mor, 2026; Gibson et al., 2026). ROS and elevated intracellular calcium activate DRP1, producing an imbalance in mitochondrial dynamics and fragmentation of the mitochondrial network. Impaired mitophagy then allows damaged mitochondria to accumulate, while ATP-deficient motor proteins fail to deliver healthy mitochondria to synaptic sites for rescue (Gabrielli et al., 2022; Rybka et al., 2019).

**FIGURE 3:**
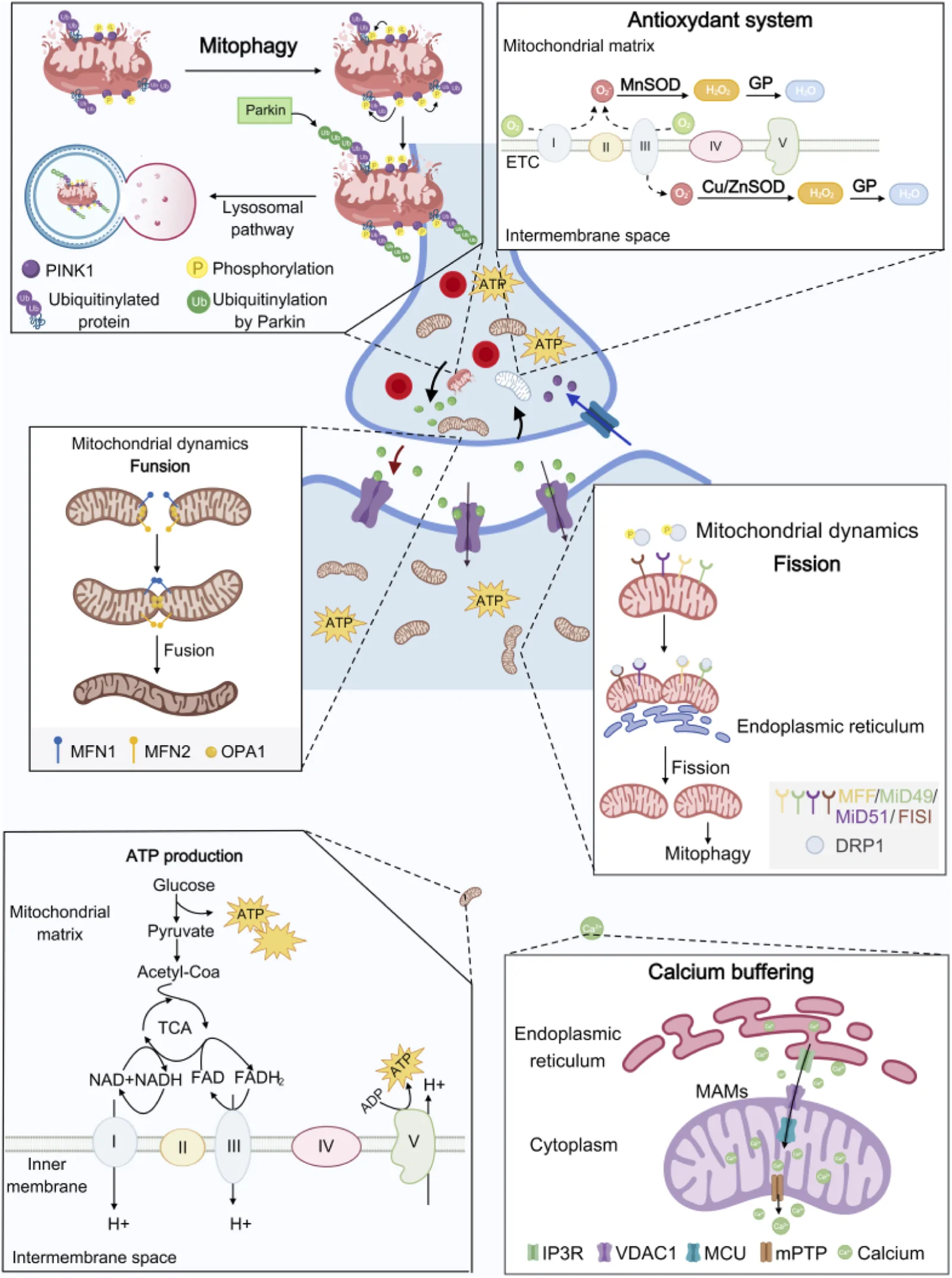
This figure summarises the multiple mitochondrial dysfunctions that converge to cause synaptic failure in AD. Chief defects include impaired mitophagy, in which damage to the PINK1/Parkin pathway prevents the removal of defective mitochondria; oxidative stress and an energy crisis, in which a weakened antioxidant system together with an abnormal ETC generate excessive ROS and reduce ATP production; excessive fission with inadequate fusion, leading to mitochondrial fragmentation; and disrupted calcium homeostasis, in which abnormal mitochondria-associated ER membranes function causes calcium overload and opening of the mitochondrial PTP. These processes interact and amplify one another, thereby compromising synaptic energy supply and stability and contributing to cognitive decline.

The consequence is progressive damage to synaptic terminals. Structurally, this damage manifests as dendritic spine atrophy and vacuolation of presynaptic terminals. Functionally, it appears as a reduced probability of neurotransmitter release and loss of synaptic plasticity, including impaired LTP and LTD. These changes culminate in synapse loss, disruption of neural circuits, and consequent cognitive or motor deficits (Zhang Y. et al., 2025; Ra et al., 2019) (Figure 3).

### 3.1 Imbalance of calcium ion homeostasis

Synaptic communication depends on the fusion of neurotransmitter-containing vesicles with the presynaptic membrane, a process triggered by Ca^2+^ influx (Südhof, 2014). In neurons, mitochondria, the endoplasmic reticulum (ER), and lysosomes constitute the organellar network that maintains Ca^2+^ homeostasis. Membrane depolarization during synaptic activity opens voltage-gated calcium channels (Yang Y. M. et al., 2025), allowing rapid Ca^2+^ entry into the presynaptic terminal and thereby initiating vesicle fusion and neurotransmitter release. Mitochondria situated near the synapse promptly take up Ca2+, raising matrix Ca^2+^ levels; this uptake buffers cytoplasmic Ca^2+^ transients and helps regulate the strength of synaptic signalling (Rueda et al., 2016; Devine and Kittler, 2018). In response to the abrupt cytoplasmic Ca^2+^ rise elicited by synaptic activity, mitochondria absorb and sequester large amounts of Ca^2+^ (Patron et al., 2014; Rizzuto et al., 2012).

The endoplasmic reticulum, the principal cellular Ca^2+^ store, conveys Ca^2+^ signals to mitochondria via membrane contact sites known as mitochondria-associated endoplasmic reticulum membranes (MAMs) (Area-Gomez et al., 2009; Markovinovic et al., 2022). The inositol 1,4,5-trisphosphate receptor at MAMs functions as a Ca^2+^ release channel (Bartok et al., 2019), mediating ER Ca^2+^ efflux and creating a local high-Ca^2+^ microdomain at the MAM interface (Csordás et al., 2010; Krols et al., 2016). Mitochondrial Ca^2+^ uptake proceeds in two stages: first, Ca^2+^ diffuses into the intermembrane space through voltage-dependent anion channels in the outer mitochondrial membrane (Shoshan-Barmatz et al., 2017); second, uptake across the inner mitochondrial membrane is effected by the MCU (Baughman et al., 2011; De Stefani et al., 2011), which remains largely inactive until cytoplasmic Ca^2+^ concentrations rise to the micromolar range (Colussi and Stathopulos, 2024; Chapoy et al., 2023). Physiological mitochondrial Ca^2+^ uptake activates key tricarboxylic acid cycle enzymes, enhancing oxidative phosphorylation efficiency, increasing local ATP production, and regulating synaptic plasticity and the release of neurotransmitters (Brito-Estrada et al., 2025; Kwon et al., 2016). In addition, mitochondrial Ca^2+^ buffering improves the frequency and spatiotemporal profile of intracellular Ca^2+^ signals, modulating downstream calcium-dependent signals.

Mitochondria located at dendritic spines and synaptic sites also play important local roles in physiological and pathological Ca^2+^ signalling. Dendritic spines, as spatially discrete microdomains of Ca^2+^ signals, contain mitochondria that provide local Ca^2+^ buffering (Smith-Dijak et al., 2019). Dendritic shortening and branch loss can occur independently of neuronal death and are closely linked to local mitochondrial Ca^2+^ handling (Hambright et al., 2017). Local rises in Ca^2+^ driven by synaptic activity promote mitochondrial anchoring near synapses, furnishing rapid buffering at the site. Synapses that lack mitochondria can show abnormally increased excitability and impaired homeostatic plasticity (Girard et al., 2012; Divakaruni et al., 2018). Overactive neurons often sustain prolonged Ca^2+^ influx, predisposing mitochondria to Ca^2+^ overload; this exhausts mitochondrial network function and leads to ROS generation, organelle damage, and removal by autophagy (Shen et al., 2019). Consequently, in neurodegenerative diseases and psychiatric disorders, neurons that have lost mitochondrial content are unusually vulnerable to excitotoxicity.

Dysregulation of mitochondrial Ca^2+^ has been shown to mediate sublethal dendritic atrophy and thus plays a key role in AD. Post-mortem studies and imaging of patients and animal models reveal characteristic dendritic simplification and atrophy (Koleske, 2013; Knopman et al., 2021; Alevriadou et al., 2021). Inhibiting excessive mitochondrial Ca^2+^ uptake via the MCU is neuroprotective (Liu et al., 2016; Hamilton et al., 2021), and MCU inhibitors confer benefit across diverse genetic models of chronic neurodegenerative disease (Márta et al., 2021; Shankar et al., 2008). These findings indicate that pathological mitochondrial Ca^2+^ handling, rather than physiological activation of cytoplasmic Ca^2+^-dependent enzymes, underlies mitochondrial failure and dendritic retraction.

AD involves a complex network of calcium regulation. Oligomeric A β , the central pathological species, exerts early and multifaceted harm. Its accumulation within synaptic mitochondria produces mitochondrial calcium overload and collapse of the membrane potential (Pizzo et al., 2020). In animal models of AD, A β raises mitochondrial calcium concentration; this effect can be reversed by MCU inhibitors, supporting a pathogenic A β –MCU–calcium overload axis. Familial AD-linked presenilin mutations perturb neuronal calcium homeostasis by a different mechanism. These mutations cause endoplasmic reticulum calcium overload and potentiate the activity of ryanodine receptors and IP3 receptors, thereby increasing calcium release (Makarov et al., 2023). Mice carrying presenilin mutations display neuronal hyperexcitability and reduced mitochondrial calcium-buffering capacity in the hippocampus. The resulting sustained hyperexcitability promotes mitochondrial calcium overload, which precipitates mitochondrial dysfunction and autophagy (Rojas-Charry et al., 2020). Of note, while the “calcium overload” hypothesis is widely supported, both upregulation and downregulation of mitochondrial Ca^2+^ uptake have been reported in different AD models carrying presenilin mutations, reflecting context-dependent effects and disease-stage-specific differences (Reddy and Beal, 2005; de Veij Mestdagh et al., 2023). This complexity should be considered when interpreting the pathophysiological role of calcium dysregulation in FAD.

Pathological tau establishes a malignant feedback loop that inhibits the mitochondrial calcium efflux pump, the mitochondrial Na^+^/Ca^2+^ exchanger (NCLX), and thereby reduces calcium clearance. Loss of NCLX accelerates pathology and memory decline in AD model animals (Jadiya et al., 2019). Calcium regulation in AD brain tissue is complex and region-specific: transcriptome analyses indicate downregulation of calcium influx genes (MCU and MICU1) with upregulation of NCLX, the latter likely a compensatory response. However, in some regions, NCLX protein levels are reduced, implying that this compensation fails in a manner dependent on disease stage and brain region (Xu et al., 2024).

Collectively, these disturbances collapse synaptic mitochondrial calcium homeostasis, precipitating dendritic degeneration and synaptic loss via opening of the mitochondrial permeability transition pore, oxidative bursts, and energy failure (Bierhansl et al., 2024). Dysregulation of synaptic mitochondrial calcium thus links upstream pathogenic factors to downstream synaptic dysfunction. Restoring mitochondrial calcium handling at key nodes therefore represents a promising neuroprotective strategy.

### 3.2 A vicious cycle of synaptic mitochondrial energy metabolism disorder and oxidative stress

Neurons are terminally differentiated cells with very high energy requirements. Energy-intensive processes such as synaptic transmission therefore rely on efficient ATP production by mitochondria. Although the brain constitutes only about 2% of body mass, synaptic activity accounts for over 20% of the body’s basal energy consumption, primarily because of this synaptic activity (Harris et al., 2012).

Mitochondria in presynaptic terminals, which support vesicle cycling and neurotransmitter release, and those in postsynaptic compartments, such as dendritic spines that receive and integrate signals, form distinct, functionally specialised subpopulations (Yang L. et al., 2025). At rest, neurons rely mainly on glycolysis for basal ATP production. However, during periods of high activity—for example, action potential propagation, synaptic vesicle cycling, restoration of ion gradients, and postsynaptic receptor signaling—neurons rapidly shift to oxidative phosphorylation (OXPHOS), making synaptic mitochondria the primary energy source under these conditions (Justs et al., 2022). Thus, the bioenergetic efficiency of synaptic mitochondria directly influences the integrity, plasticity, and stability of synaptic function.

The primary concern concerning synaptic mitochondrial energy metabolism is the low efficiency of OXPHOS, due to local calcium overload due to abnormal synaptic activity and the collapse of the mitochondrial membrane potential. Furthermore, damage to proteins or downregulation of ETC complexes, especially in synaptic mitochondria, can increase this problem (Vaillant-Beuchot et al., 2021). Within the synaptic microdomain, activity of key tricarboxylic acid cycle enzymes that supply reducing equivalents to synaptic mitochondria is diminished, causing a local energy shortfall at synapses. Because synaptic activity demands large amounts of ATP—for vesicle loading, release, and recycling, and for Na^+^/K^+^-ATPase and Ca^2+^-ATPase activity to maintain ion gradients across the synaptic cleft—the energy output of synaptic mitochondria is insufficient. Consequently, neurotransmitter release becomes rapid, recovery of the postsynaptic membrane potential is delayed, and synaptic Ca^2+^ buffering capacity is reduced. Altogether, these alterations compromise the precision and stability of synaptic transmission (Pathak et al., 2015). Synaptic mitochondria are the principal source of ROS within the synaptic microdomain. Under physiological conditions, approximately 1%–2% of electrons in the ETC “leak” to form superoxide anions at complexes I and III (Jomova et al., 2023). Monoamine oxidase in the outer membrane of synaptic mitochondria generates hydrogen peroxide while degrading neurotransmitters such as dopamine and serotonin. Although neurons possess antioxidant defense mechanisms, synaptic microdomains—particularly those distant from the cell body—have limited capacity to synthesize, regenerate, and replenish antioxidants such as glutathione (GSH) (Baxter and Hardingham, 2016). If the electron transport chain in synaptic mitochondria is impaired—increasing electron leakage—or if antioxidant defences are weakened, ROS production can exceed local clearance. This disrupts redox homeostasis and allows local oxidation to directly damage synaptic proteins, lipids, and mitochondrial membranes.

Energy crisis and oxidative stress are closely intertwined and directly linked to synaptic mitochondrial dysfunction and disease progression. Synaptic mitochondrial function is especially critical and closely linked to disease progression. Positron emission tomography reveals focal synaptic glucose hypometabolism in early-stage AD brains (Croteau et al., 2018). Reduced concentrations of tricarboxylic acid cycle intermediates and lower lactate levels in cerebrospinal fluid further indicate impaired oxidative metabolism (Trushina et al., 2013).

A β oligomers are the predominant pathogenic species in AD: they form at synapses, bind to mitochondrial proteins, and inhibit electron transport chain complexes, disrupt mitochondrial Ca^2+^ handling, and generate excessive ROS (Hammond et al., 2020). ROS from synaptic mitochondria in turn activate synaptic signaling pathways that further promote amyloidogenesis and other forms of synaptic dysfunction. ROS also induce β-site amyloid precursor protein cleaving enzyme 1 (BACE1) activity, increasing A β production, and activate kinase cascades such as p38 mitogen-activated protein kinase (p38 MAPK) and c-Jun N-terminal kinase (JNK), which drive tau hyperphosphorylation, compromise microtubule stability, and impair synaptic transport (Bai et al., 2022).

Thus, A β and tau pathology, synaptic mitochondrial dysfunction, and oxidative stress form an interrelated pathological axis. Notably, oxidative stress and its synaptic biomarkers appear before the formation of A β plaques and neurofibrillary tangles (Park et al., 2021).

Excessive ROS damage synaptic proteins, lipids, and mitochondrial DNA (Jové et al., 2021). Oxidative stress also damages synaptic mitochondrial biogenesis regulated by peroxisome proliferator-activated receptor coactivator 1 (PGC-1) and downstream factors (You et al., 2024). In AD models, the PGC-1α signalling pathway in synaptic mitochondria is often suppressed, impairing mitochondrial renewal. Consequently, damaged synaptic mitochondria are not effectively replaced, producing local energy deficits and leading to synaptic structural degeneration (Peng et al., 2017).

Chronic cerebral hypoperfusion and intermittent hypoxia deplete the enzymatic sources of ROS, impair synaptic function, and accelerate Alzheimer’s disease (Sorrentino et al., 2018; Peng et al., 2020). Synaptic mitochondrial energy metabolism and local oxidative stress are the main drivers of early synaptic dysfunction, dendritic spine loss, and AD cognitive decline. Targeting synaptic mitochondria in this vicious cycle (for example, improving bioenergetics, strengthening antioxidant defenses, or promoting healthy mitochondrial turnover) is a promising treatment strategy to maintain synapses and slow AD progression.

### 3.3 Imbalance of synaptic mitochondrial dynamics

Neurons are highly polarized, complex cells that require precise spatiotemporal distribution of mitochondria within axons and dendrites to maintain functional homeostasis. Mitochondrial dynamics—comprising cytoskeletal transport and morphological remodelling by fusion and fission—ensure that synapses receive energy and Ca^2+^ buffering on demand (Demarest et al., 2020). In AD, this tightly regulated system is disrupted early at synapses (Tang et al., 2019). Consequently, synaptic mitochondrial dysfunction constitutes an upstream pathology that precipitates synaptic failure, increases oxidative stress, and ultimately leads to synapse loss.

#### 3.3.1 Precise regulation of mitochondrial transport and synaptic localization

Synaptic mitochondria rely on the microtubule cytoskeleton and motor proteins for long-range, bidirectional transport in neurons. Kinesin motors drive anterograde movement to presynaptic terminals, while dynein mediates retrograde transport toward the soma. As described in Section 2.2, the Miro1/2–TRAK1/2 complex regulates motor attachment to the mitochondrial outer membrane (Li et al., 2015; Hu et al., 2020). When synaptic activity raises local calcium, calcium binding to Miro1/2 causes motor proteins to disengage from mitochondria, enabling motile mitochondria to pause and anchor at active synapses where energy demand and calcium signaling are highest.

#### 3.3.2 Specific regulation by fusion and fission of synaptic mitochondrial function and positioning

Mitochondria are remodeled by opposing fusion and fission processes, both vital for synaptic stability and plasticity. Fusion by Mitofusin 1/2 (MFN1/2) and OPA1 restores damaged regions and homogenizes metabolic function; fission by DRP1 separates damaged material and produces smaller organelles that can enter narrow postsynaptic compartments such as dendritic spines (Zhang et al., 2019). The relative balance between the two processes directly impacts synaptic development and function. Inhibition of Drp1-mediated fission in hippocampal neurons results in excessive mitochondrial elongation, preventing them from entering dendritic spines and ultimately reducing synaptic density. However, a moderate increase in fission increases mitochondrial localization to spines and spine density (Cheng et al., 2022).

Most importantly, this morphological regulation is compartment-specific. In neurons with downregulated fission receptor Mitochondrial Fission Factor (MFF)—a key outer mitochondrial membrane receptor that recruits the fission GTPase Drp1 to drive mitochondrial division—presynaptic mitochondrial volume is selectively increased, and calcium uptake capacity is increased, buffering presynaptic calcium transients and modulating neurotransmitter release, while dendritic mitochondrial morphology and basal ATP production remain unchanged (Lee et al., 2018). These results indicate that different synaptic microdomains tune mitochondrial morphology to meet local functional needs.

A β oligomers and hyperphosphorylated tau impair synaptic mitochondrial dynamics. Oligomers destabilize Miro1 and reduce its calcium-sensing capacity and cause defects in presynaptic mitochondrial transport (Yao et al., 2022). Both Aβ oligomers and hyperphosphorylated tau activate Drp1 and promote proteolysis of OPA1, leading to excessive mitochondrial fragmentation (Kandimalla et al., 2018). The imbalance of fission and fusion causes larger, functionally weak mitochondria at synapses, which generate less ATP and accumulate locally due to a lack of clearance. Excessive ROS production from dysfunctional synaptic mitochondria leads to synaptic energy failure and oxidative stress, which leads to dendritic spine atrophy and loss of synapses (Joshi et al., 2019).

### 3.4 Dysfunction of mitophagy in synaptic terminals

Mitophagy is a selective quality control process to remove damaged mitochondria. In neurons, the removal of dysfunctional mitochondria at synaptic sites (synaptic mitophagy) keeps metabolism and calcium homeostasis in high-energy, high-oxidative-stress compartments (Soubannier et al., 2012). The removal of synaptic mitochondria damaged by high-frequency activity, calcium overload, or oxidative stress prevents the accumulation and propagation of secondary toxic events (Roberts et al., 2021).

The PINK1–Parkin pathway is the most important tool used to monitor mitochondrial quality at neuronal synapses. When mitochondrial membrane potential is lost, PINK1 stabilizes on the outer membrane and activates Parkin, initiating ubiquitin-dependent modification and autophagosomal engulfment (Sliter et al., 2018). In neurons, this pathway controls the spatial clearance and redistribution of synaptic mitochondria.

For example, following Parkin activation, the synaptic mitochondrial adaptor Miro is selectively ubiquitinated and degraded, causing damaged mitochondria to detach from microtubule tracks. This halts their transport to synapses and permits replacement by healthy organelles (Birsa et al., 2014). In postsynaptic compartments such as dendritic spines, timely mitophagy removes dysfunctional mitochondria; this is essential for maintaining intraspine metabolism and local protein synthesis and for regulating α-amino-3-hydroxy-5-methyl-4-isoxazolepropionic acid (AMPA) receptor membrane trafficking, thereby influencing synaptic plasticity (Reytor-González et al., 2025). Impaired synaptic autophagy leads to the selective accumulation of dysfunctional mitochondria in dendritic spines; excess ROS oxidatively damage postsynaptic density proteins (PSD-95, a key scaffolding protein at excitatory synapses that organizes glutamate receptors and signaling molecules) and ultimately lead to spine atrophy and loss of function (Fang et al., 2019).

Synaptic mitophagy is severely impaired in AD. A β and hyperphosphorylated tau bind together to disrupt the PINK1–Parkin pathway, increasing synaptic mitochondrial damage, inhibiting Parkin recruitment or its ubiquitination activity, and reducing autophagosome–lysosome fusion efficiency (Pradeepkiran et al., 2025). A large number of dysfunctional ROS-generating mitochondria accumulate in presynaptic terminals and dendritic spines, which not only do not provide bioenergetic support but also localize oxidative stress and inflammatory signalling, damaging synaptic proteins and lipids and accelerating synapse loss (He et al., 2019). Synaptic regions in AD patients and model animals express abnormal mitophagy markers and blocked autophagic flux; mitochondrial accumulation in these regions correlates with loss of synaptic density and cognitive decline (Mary et al., 2023). Selectively restoring mitophagic flux in synaptic compartments decreases synaptic injury and improves cognition in AD models (Zhou et al., 2025).

### 3.5 Integrative molecular feedback loops linking calcium dysregulation, bioenergetic failure, dynamic imbalance, and impaired mitophagy at the synapse

The four pathological processes described in Sections 4.1–4.3 do not operate in isolation; they are mechanistically intertwined and constitute self-reinforcing vicious cycles that accelerate synaptic demise in Alzheimer’s disease. Here, we explicitly delineate the principal molecular feedback loops that connect them.

Calcium–energy–ROS axis. Synaptic activity and A β oligomers drive excessive mitochondrial Ca^2+^ uptake via MCU, which collapses the mitochondrial membrane potential, inhibits electron transport chain complexes, and curtails ATP synthesis. The resulting ATP deficit impairs the activity of plasma membrane Ca^2+^-ATPases and Na^+^/K^+^-ATPases, slowing Ca^2+^ clearance and further aggravating mitochondrial calcium overload. The ETC dysfunction simultaneously increases electron leakage at complexes I and III, generating superoxide and other ROS. These ROS oxidize iron–sulfur clusters, ETC subunits, and membrane lipids, further suppressing oxidative phosphorylation and amplifying energy failure—a classical feedforward loop.

Dynamics–mitophagy–ROS loop. Elevated matrix Ca^2+^ and ROS activate the fission GTPase DRP1 and inhibit fusion proteins, promoting excessive mitochondrial fragmentation. Fragmented synaptic mitochondria are bioenergetically less efficient and often fail to be properly transported into dendritic spines and presynaptic terminals, creating local ATP deficits. Moreover, severely fragmented mitochondria with low membrane potential frequently escape PINK1/Parkin-mediated recognition, yet they continue to leak ROS. Hyperphosphorylated tau exacerbates the situation by inhibiting the mitochondrial Na^+^/Ca^2+^ exchanger NCLX and disrupting autophagosome–lysosome fusion, thereby blocking clearance. The resultant accumulation of ROS-generating mitochondria at synapses sustains oxidative stress and lipid peroxidation, which in turn activate stress kinases that promote both tau hyperphosphorylation and BACE1-mediated A β production, linking back to the initiating insults.

Calcium–mitophagy crosstalk. Sustained mitochondrial Ca^2+^ overload impairs autophagic flux by disrupting lysosomal acidification and by activating calpains that cleave key autophagy proteins. Impaired mitophagy permits the persistence of dysfunctional mitochondria that continuously release Ca^2+^ and ROS, perpetuating calcium dysregulation and providing a direct link between defective clearance and sustained calcium pathology.

Convergence of upstream pathology. A β oligomers and pathological tau simultaneously strike multiple nodes of this network: A β directly inhibits ETC complexes and enhances MCU-dependent Ca^2+^ uptake, while tau inhibits NCLX, compromises retrograde transport of autophagosomes, and blocks lysosomal fusion. Thus, an initial insult at any one of the four nodes—be it calcium overload, oxidative stress, mitochondrial fragmentation, or mitophagy failure—can propagate through these interconnected loops to recruit the others. This interconnectivity explains the relentless and synergistic progression of synaptic failure observed in AD.

Therapeutic implications. The existence of these reciprocal positive-feedback loops suggests that targeting a single mechanism in isolation may be insufficient to halt synaptic deterioration. Rational combination therapies that simultaneously address complementary nodes (an antioxidant combined with a mitophagy enhancer, or an MCU inhibitor together with a DRP1 inhibitor) are more likely to break the vicious cycle at the synapse and are therefore a central requirement for the translational framework outlined in Section 6.

## 4 The central significance of synaptic mitochondrial dysfunction in AD

Mitochondrial dysfunction at synapses is a critical node in the AD network. It precedes the formation of hallmark amyloid plaques and neurofibrillary tangles and directly contributes to synaptic failure and cognitive decline (Cai and Tammineni, 2017; Cai et al., 2011). Recognizing synaptic mitochondria as this key locus is essential for understanding AD pathogenesis and for developing novel therapies.

### 4.1 Synaptic mitochondrial dysfunction is an early and central driving event in AD

There is substantial evidence that synaptic mitochondrial dysfunction is an early, central, and detectable feature of AD. Proteomic analyses show that, early in the disease, proteins of synaptic mitochondria involved in oxidative phosphorylation and the tricarboxylic acid cycle undergo selective alterations (Torres et al., 2021). At synapses, A β oligomers and hyperphosphorylated tau preferentially localize around mitochondria, thereby compromising their function via diverse pathological mechanisms (Lu et al., 2023). A β promotes mitochondrial Ca^2^ overload at synapses, inhibits electron transport chain complex activity, and triggers local bursts of reactive oxygen species, which impair mitochondrial transport to synapses and thereby reduce the local energy supply (Cardanho-Ramos et al., 2024; Pradeepkiran et al., 2024).

Consequently, local ATP production falls markedly, redox homeostasis is disturbed, and calcium buffering is severely impaired. Together, these defects disrupt presynaptic vesicle cycling and compromise postsynaptic receptor function, ultimately causing dendritic spine loss (Spires-Jones and Hyman, 2014).

### 4.2 Malignant cycle of synaptic mitochondrial dysfunction, pathological proteins, and synapse loss

Dysfunction of synaptic mitochondria and AD form a mutually exacerbating cycle within the synaptic microenvironment. The relationship between synaptic mitochondrial dysfunction and proteinopathies is bidirectional and self-reinforcing. A β and tau damage synaptic mitochondria, while excessive ROS released from these mitochondria activate proteolytic pathways that increase Aβ production and kinases that drive tau hyperphosphorylation (Pradeepkiran et al., 2022). Synaptic mitochondria also undergo structural disruption: Drp1-mediated excessive fission and impaired fusion produce mitochondrial fragmentation at synapses (Rangaraju et al., 2019). This fragmentation impairs local energy metabolism and disrupts mitophagy pathways, reducing PINK1/Parkin-mediated clearance; as a result, damaged mitochondria are not removed effectively, and injurious changes persistently accumulate (Pradeepkiran et al., 2024).

Together, these events—pathogenic proteins damaging synaptic mitochondria, failure of mitochondrial function and quality control, synaptic energy crisis and oxidative stress, and loss of synaptic structure—are the major culprits for progressive cognitive decline in AD (Wang et al., 2023).

### 4.3 Synaptic mitochondria as a critical target for early therapeutic intervention

Synaptic mitochondrial dysfunction precedes widespread neuronal loss and is therefore an important, potentially reversible window of treatment for AD (Picard et al., 2018; Kerr et al., 2017). Protecting synaptic mitochondrial function has become a central goal of promising neuroprotective strategies (Reddy and Oliver, 2019). Selectively improving synaptic energy supply, antioxidant defenses, and quality control will increase synaptic resilience, counteract the toxic effects of upstream pathogenic proteins, preserve synaptic structure and function, and slow the progression of cognitive decline (Sharma et al., 2021). Current intervention strategies are shifting toward multidimensional approaches that directly target the synaptic microenvironment to control synaptic metabolic processes, oxidative stress, quality control, and energy metabolism. These approaches fall into three broad categories. First, new small molecules and biological agents—for example, mitochondria-targeted antioxidant peptides and modulators of metabolic pathways—are being developed to correct local biochemical imbalances within synapses (Manczak et al., 2010). Second, interventions targeting cerebral metabolism are used to remodel brain energy use (Cunnane et al., 2016). Third, endogenous molecules and natural compounds are studied for their multi-target neuroprotective effects (Calabrese et al., 2009). All of these methods aim at the end goal of restoring synaptic function. By enhancing synaptic resilience as a defense mechanism, they represent promising avenues for altering Alzheimer’s disease progression (Mattson and Arumugam, 2018).

## 5 Therapeutic strategies for AD targeting synaptic mitochondrial dysfunction

As AD has become more understood, intervention strategies have moved from simply removing pathological proteins to targeting upstream processes that drive disease progression. Synaptic mitochondrial dysfunction (calcium imbalance, energy failure, oxidative stress, dysregulation, and autophagy) is now recognized as the major driver of early synaptic loss and cognitive impairment (Pal, 2025; Wang S. et al., 2025).

Thus, therapeutic strategies that protect, repair, or enhance synaptic mitochondrial function are promising avenues for delaying or even reversing AD. In this section, we review intervention strategies classified by mechanism of action and discuss their limitations.

### 5.1 Strategies targeting energy metabolism and oxidative stress

Small molecules and peptides: The mitochondria-targeted antioxidant peptide SS-31 (Elamipretide) accumulates at the inner mitochondrial membrane, scavenges mitochondria-derived ROS, and protects cardiolipin from oxidation. In AD mice, SS-31 lowers oxidative stress in synaptic mitochondria, restores their dynamics and function, and preserves synaptic integrity independently of A β plaque burden (Zhao et al., 2019). Mild inhibitors of mitochondrial complex I (mtCI), such as C458, activate AMPK signaling through partial inhibition of mtCI (Stojakovic et al., 2021). In AD models, this reduces ROS surges, stimulates mitochondrial biogenesis, and dampens mitochondrial apoptotic pathways. Novel bifunctional small molecules provide additional advantages; for example, B14 reduces A β accumulation and inhibits the mitochondrial apoptotic pathway by stabilizing the B-cell lymphoma 2 (Bcl-2) promoter while improving the ultrastructure and antioxidant capacity of synaptic mitochondria (Cummings et al., 2024). KDS12025, a small-molecule compound with hemoglobin pseudoperoxidase-mimetic activity, enhances hemoglobin pseudoperoxidase activity, efficiently decomposes hydrogen peroxide, and reduces astroglial oxidative stress in AD models, thereby preserving synaptic mitochondrial function (Guo et al., 2022). However, clinical translation remains difficult. SS31 has shown promise in improving mitochondrial function in trials of hereditary mitochondrial disease, but it has not advanced to a pivotal clinical stage in AD.

Clinical data for Mitochondria-targeted Ubiquinone (MitoQ) in AD are likewise limited and inconclusive. Several factors may account for these failures or the current stagnation. First, oxidative damage in the AD brain is spatiotemporally heterogeneous, so simple antioxidant therapies may address only downstream symptoms rather than primary causes. Second, the absence of biomarkers that dynamically and quantitatively report the redox status of synaptic mitochondria *in vivo* prevents precise enrollment of patients with a “synaptic mitochondrial oxidative stress” phenotype and hinders demonstration of target engagement. Third, interventions may be given too late: after substantial neuronal loss, protecting the remaining synapses may be insufficient to yield measurable improvements in global cognition.

Metabolic reprogramming may be promising therapeutics. For example, the ketogenic diet induces physiological ketosis in the brain and provides beta-hydroxybutyrate as fuel for ATP production. This fuel directly supports mitochondrial ATP production when mitochondrial function and antioxidant capacity are compromised. Preclinical studies have shown that diet can improve cognitive function (Kashiwaya et al., 2013).

Drug repurposing has also shown metabolic modulation strategies. Anti-diabetic metformin activates the AMPK pathway, promotes mitochondrial biogenesis, improves energy metabolism, and reduces ROS production, effectively reducing synaptic damage in AD models (Samaras et al., 2020). Glucagon-like peptide-1 receptor agonists like liraglutide can also be used to restore synaptic mitochondrial function in AD mice and reduce oxidative stress and proteinopathy-related damage (Tang et al., 2025). Hyperbaric oxygen therapy increases cerebral tissue oxygenation and has been shown in animal models to improve mitochondrial respiratory function and reduce oxidative stress. Preclinical studies show cognitive improvements, likely due to relief of cerebral energy metabolism deficit (Lin et al., 2024). The main clinical problems of these drugs are poor long-term adherence, marked variability in metabolism among individuals, and a lack of standardized protocols to turn treatment into an optimal “ketotic” time. Drug repurposing has shown neuroprotective effects in animals, but large clinical studies in nondiabetic AD populations have not shown the results, and the optimal dose and time of intervention are not known.

Endogenous hormones and nutrients: Melatonin is an endogenous antioxidant targeting mitochondria, neutralizing free radicals, maintaining mitochondrial membrane potential, removing damaged mitochondria by mitophagy, and preserving synaptic function in several preclinical AD models (Bocheva et al., 2024). Lutein stimulates mitochondrial biogenesis via the PGC1/NRF1/TFAM pathway and significantly increases antioxidant enzyme activity in AD models (He et al., 2023). Fibroblast growth factor 21 (FGF21) boosts the antioxidant capacity of synaptic mitochondria and suppresses neuroinflammation by AKT and AMPK activation (Zhao et al., 2026).

The translation of endogenous hormones and nutrients is limited to clinical applications. First, physiological concentrations are often far from therapeutic doses needed for neuroprotection; orally administered melatonin lacks stable, effective brain concentrations and can disrupt physiological rhythms. Second, key protein factors such as FGF21 are delivered as a single injection. They require repeated injections and very poor targeting intracranially, causing low patient compliance and limited therapeutic effect. Third, these drugs act pleiotropically. Without biomarkers for “synaptic mitochondrial dysfunction,” it is impossible to identify the patients most likely to benefit from them, which severely diminishes efficacy signals in large heterogeneous trials.

Active components of traditional Chinese medicine: Some bioactive compounds from traditional Chinese medicinal herbs have potential for modulating synaptic mitochondrial function because they target multiple targets and pathways. For instance, ginsenoside Rk1 restores A-induced collapse of mitochondrial membrane potential, lowers intracellular ROS, and suppresses neuronal apoptosis (She et al., 2024). Resveratrol activates the AMPK/SIRT1/PGC1 axis, improves mitochondrial biogenesis, increases energy metabolism, reduces oxidative stress, and modulates the synaptic microenvironment (Griñán-Ferré et al., 2021). While there are clear preclinical mechanisms, most large clinical trials have not met primary endpoints. One reason is variability in herbal sources and extraction methods, which produces uncontrolled contents and ratios of active compounds and fails to meet modern pharmaceutical standards for “consistent quality and batch-to-batch stability.”

### 5.2 Strategies targeting calcium homeostasis dysregulation

Small changes in neuronal Ca^2+^ homeostasis are early and decisive factors in synaptic mitochondrial dysfunction in AD (Calvo-Rodriguez et al., 2020). As discussed in Section 3.1, pathological calcium overload, Ca^2+^ entry via the MCU, loss of endoplasmic reticulum–mitochondria contact sites, or overactivation of glutamate receptors such as N-methyl-D-aspartate (NMDA) receptors directly damages mitochondria and leads to synaptic failure (Ashrafi et al., 2020). Restoring calcium homeostasis is one of the main strategies to maintain synapses and slow AD progression. This section reviews interventions that directly or indirectly target pathological calcium signaling pathways.

Small molecules and proteins: Certain inhibitors selectively target calcium channels or transporters. For example, Ru265 and MCUi4, selective inhibitors of the MCU, block excessive calcium entry into mitochondria and thereby prevent calcium-overload-induced neuronal death and mitochondrial collapse in AD models (Woods et al., 2019). Acting further downstream in the calcium signaling pathway, the antibiotic ceftriaxone reduces overactivation of NMDA receptors and the resultant toxic calcium influx by upregulating glutamate transporter 1 (GLT-1) in astrocytes. This decreases synaptic glutamate accumulation and improves synaptic mitochondrial function and cognition in AD models (Hameed et al., 2019).

Some recent strategies aim to block several calcium entry routes at once. For example, NMDA receptor antagonists combined with synthetic peptides that inhibit the pore-forming activity of Aβ oligomers can almost completely abolish Aβ-induced neuronal calcium responses (Caballero et al., 2022). Concerning aberrant calcium transfer at mitochondria-associated endoplasmic reticulum membranes (MAMs), ApoE4 (the major genetic risk factor for AD) modulates ER-to-mitochondria Ca^2+^ transfer through upregulation of the molecular tether GRP75. Notably, GRP75 upregulation enhances the efficiency of Ca^2+^ delivery from the ER to mitochondria, which may exacerbate mitochondrial calcium overload under pathological conditions (Liang et al., 2021). Thus, strategies aimed at normalizing MAM-mediated calcium transfer—rather than simply inhibiting GRP75—may offer a more refined approach to restore calcium homeostasis at this junction.

Significant treatment challenges remain. No MCU inhibitors have been evaluated for AD, largely because MCU fulfills essential physiological roles in peripheral organs such as the heart; systemic inhibition therefore risks serious cardiovascular side effects. A pragmatic strategy is not to abandon the target but to develop agents with restricted central nervous system distribution (for example, ligands exploiting brain-specific transporters) or to pursue safer approaches that modulate MCU assembly rather than block its pore. Early clinical studies have reported some safety signals and hints of cognitive benefit, but the outcomes of definitive phase III trials remain unknown. A further difficulty is achieving subunit selectivity: even when targeting specific NMDA receptor subunits, it is hard to distinguish synaptic from extrasynaptic signaling perfectly.

Metabolic and organelle-function modulators: stabilizing calcium homeostasis indirectly by altering metabolism or inter-organelle communication. Modulators that act on metabolism or organelle function stabilize calcium homeostasis indirectly by altering cellular metabolism or communication between organelles. For example, the natural antioxidant astaxanthin reduces excitotoxic calcium influx by modulating glutamate receptor activity and increases the antioxidant capacity of downstream mitochondria (Kandy et al., 2022). Another strategy targets the mitochondrial permeability transition pore (mPTP), which represents the terminal pathway of mitochondrial injury triggered by calcium overload. Despite these promising approaches, the field remains nascent. A principal challenge is the design of small molecules that selectively regulate protein–protein interactions within the dynamic microarchitecture of MAMs without impairing the autonomous functions of either the endoplasmic reticulum or mitochondria. To address this, multifunctional nanoparticles have been explored; for instance, a “nanobreak” co-displays magnesium ions together with siRNA against the mPTP regulator CypD.

In an AD mouse model, the strategy prevented mitochondrial calcium overload and mPTP opening, producing a marked improvement in synaptic mitochondrial function (Zhang et al., 2023). Restoring Wnt signaling—for example, by supplementing Wnt2b—has also been reported to improve neuronal calcium homeostasis and mitochondrial performance, likely via several cytoprotective mechanisms (Xu et al., 2023). Translation to the clinic, however, faces the common challenges of nanomedicine, including long-term biosafety, nanoparticle immunogenicity, and scalable manufacturing, and it remains unclear at which stage of disease progression inhibition of the mPTP would be most effective.

Active constituents and compound prescriptions of traditional Chinese medicine (TCM) offer several advantages for systemic regulation of calcium homeostasis. For example, serum containing the classical formulation Danggui Shaoyao San modulates calcium/calmodulin-dependent protein kinase II signalling, reduces mitochondrial calcium influx, stabilizes mitochondrial membrane potential, and inhibits neuronal apoptosis (Zhang K. X. et al., 2025). Likewise, the tonic agents Guiban Jiao and Lujiao Jiao prevent intracellular calcium overload and reactive oxygen species bursts, and they regulate the balance of mitochondrial fission and fusion proteins (Drp1 and OPA1) to preserve mitochondrial integrity (Cheng et al., 2021).

Other studies show that toxic A aggregates catalyzed by transglutaminase 2 (TG2) disturb calcium homeostasis, while Z-DON (a peptidomimetic, irreversible inhibitor of TG2 that covalently modifies its active site) can reduce calcium overload and synaptic damage in AD models (Panes-Fernandez et al., 2023). Clinical translation of these approaches faces common challenges: therapeutically effective concentrations of active components in the brain remain difficult, and there are no clinical trials stratifying AD patients by the “calcium homeostasis dysregulation” biomarker.

Targeting the vascular system and the systemic microenvironment: Alzheimer’s disease is closely linked to dysfunction of the neurovascular unit, which impairs cerebral blood flow and local metabolism and provokes neuronal calcium stress. Blocking the pathological interaction between A β -derived diffusible ligands and the α3 subunit of Na^+^/K^+^-ATPase on cerebrovascular endothelial cells has been shown to restore cerebrovascular vasodilation and, indirectly, to mitigate neuronal calcium dysregulation and mitochondrial oxidative stress (Sasahara et al., 2021). However, in advanced stages of AD, restoring vascular function alone is insufficient to reverse the established, self-sustaining neurodegenerative processes.

Preclinical work usually uses young animal models with relatively healthy vasculature, which does not recapture the structural cerebrovascular lesions seen in elderly patients. This approach also lacks real-time quantitative biomarkers for target validation and identification of the “vascular” patient subgroup, and the optimal intervention window may lie long before symptom onset, beyond conventional clinical-trial designs.

Therapeutic strategies addressing dysregulated calcium homeostasis fall into four main categories: direct inhibition of specific calcium channels, modulation of organelle interactions and metabolic state, systemic traditional Chinese medicine interventions, and cerebrovascular protection targeting central calcium signaling disturbances that contribute to synaptic mitochondrial injury. Collectively, these approaches form a primary therapeutic pathway intended to intercept the synaptic pathological cascade of Alzheimer’s disease at its origin.

### 5.3 Strategies for regulating synaptic mitochondrial dynamics

Mitochondrial networks are crucial for the maintenance, trafficking, and function of synaptic mitochondria. As noted in Section 3.3, excessive Drp1-mediated fission combined with impaired MFN2/OPA1-mediated fusion fragments the network and undermines synaptic energy supply and calcium-buffering capacity. Restoring the balance between fission and fusion therefore reinstates mitochondrial network integrity and function and recovers synaptic structure and performance.

Small molecules and peptides: Direct inhibition of the key fission protein Drp1 is the most common strategy. Mdivi-1 reverses excessive mitochondrial fragmentation in AD by inhibiting Drp1 GTPase activity, restores synaptic mitochondrial distribution and function, and reduces cognitive impairments (Su et al., 2023). Mitochondrial dynamics can also be modulated indirectly by increasing mitochondrial antioxidant capacity. SS-31 scavenges ROS and downregulates Drp1 and its receptor Fis1 in AD models while stimulating mitochondrial fusion and thus improving network morphology. MitoQ indirectly improves mitochondrial dynamics and maintains synaptic function by reducing oxidative stress (Bhatti et al., 2023). No specific Drp1 inhibitors have entered clinical trials in AD, suggesting the need for central nervous system (CNS) or neuron-selective compounds or intermittent doses to minimize systemic side effects.

Gene therapy and biologics: Gene therapy can regulate dynamic proteins precisely. One promising strategy targets the MST1/PGC1 axis: MST1 knockdown relieves the repression of PGC1. This causes mitochondrial energy metabolism genes to be activated, reduces oxidative stress, and ultimately improves synaptic function and cognition in 5xFAD mice (Cui et al., 2024). Translation is a difficult task. Its main advantage is that it can sustain benefit from one single treatment. Its main advantage is that it can sustain benefit from one treatment. The downsides include: 1) Immunogenicity and delivery efficiency: AAV vectors can induce an immune response, and a safe, efficient intracerebral delivery is needed; 2) irreversibility and risks: Long-term transgene expression complicates dose control and reverse effects; 3) Stage-dependent applicability: In late disease, many neurons are already nonfunctional, so gene therapy may be too late. Hence, mitochondrial dynamics-targeted gene therapy for AD remains at the preclinical concept validation stage.

### 5.4 Strategies for enhancing synaptic mitochondrial mitophagy

Peptides and indirect regulatory strategies: In addition to direct activation of core autophagy pathways, indirectly controlling mitochondrial damage can help quality control. As mentioned above, the mitochondria-targeted antioxidant SS-31 can lower the burden on the autophagy system by reducing the initial mitochondrial damage caused by ROS. Stabilizing mitochondrial dynamics also keeps a healthier mitochondrial network and thus facilitates autophagic clearance (Panes et al., 2023). Recent studies also show that hyperbaric oxygen therapy (HBOT) improves AD pathology through several complementary mechanisms: upregulation of the Aβ clearance receptor LRP1, activation of PINK1/Parkin-mediated mitophagy, preservation of synaptic integrity, and modulation of microglial inflammatory responses (Yao et al., 2026).

Natural product inducers: Several natural products induce mitochondrial dynamics via multiple targets. For example, apigenin acts as an MFN2 inducer, promotes outer mitochondrial membrane fusion, and improves the ultrastructure of synaptic mitochondrial cristae, thereby being neuroprotective (Tur et al., 2020). These results indicate that restoring balance between fusion and fission at multiple levels can improve synaptic mitochondrial function in AD.

Urolithin A is a well-characterized inducer of mitophagy. In cellular and animal models of AD, urolithin A activates the PINK1/Parkin pathway, clears synaptic mitochondria damaged by A toxicity, restores synaptic mitochondrial function, increases synaptic density, and, ultimately, ameliorates cognitive decline (Ho et al., 2024).

Its therapeutic action thus implies active “cleaning” of the synaptic microenvironment. Loganin also enhances mitophagy by promoting recruitment of the autophagy receptor OPTN and improves synaptic plasticity in AD (Zhou et al., 2023). Small, well-developed clinical trials in AD have been carried out. The key question is whether systemically administered autophagy inducers have enough potency and brain selectivity to preferably clear damaged mitochondria.

## 6 Conclusions and prospects

AD is a systemic failure manifesting at the synaptic level. In this review, we argue that synaptic mitochondrial dysfunction is the main factor and amplifier of this failure. A β and tau pathologies act independently, but converge on synaptic mitochondria as common targets. By altering calcium homeostasis, oxidative phosphorylation, mitochondrial dynamics, and quality control, they initiate a self-sustaining vicious cycle that subverts the energetic and molecular foundations of synaptic plasticity and turns molecular disturbances into circuit dysfunction and cognitive decline. Synaptic mitochondria are no longer merely a cellular power station but the hub where pathological signals converge, are amplified, and determine the survival of the synapse.

A new therapeutic approach has thus emerged: directly strengthening synaptic mitochondria’s functional resilience in order to create an intrinsic defense against pathological insults. It is well established, from preclinical models and clinical observations, that preserving this cellular energy system can buffer upstream toxicity and protect synaptic structure and function. This represents a shift from passive removal of pathological products to active reinforcement of neuronal homeostasis and metabolic adaptation.

However, a neuron-centric view of synaptic mitochondrial health is incomplete. The synaptic microenvironment is profoundly shaped by non-neuronal cells—most notably microglia and astrocytes—whose own mitochondrial function is intimately linked to synaptic integrity. We discuss this emerging landscape below.

### 6.1 Glial mitochondria and the neurovascular unit: reshaping the synaptic microenvironment in AD

Growing evidence indicates that mitochondrial dysfunction in microglia and astrocytes, as well as in other components of the neurovascular unit, actively contributes to the synaptic pathology observed in AD. Here, we highlight two interconnected themes: the cell-autonomous effects of glial mitochondrial failure on the synaptic microenvironment, and the recently discovered phenomenon of intercellular mitochondrial transfer.

Microglial mitochondrial dysfunction and synaptic health. Microglia, the brain’s resident immune cells, constantly survey the synaptic environment. Their surveillance, phagocytic activity, and cytokine release are all energetically demanding processes that depend on mitochondrial oxidative phosphorylation. In AD, microglia exhibit pronounced mitochondrial bioenergetic deficits, increased ROS production, and impaired mitophagy, which drive a shift toward a chronically activated, pro-inflammatory phenotype. This sustained neuroinflammation directly damages synaptic structures, inhibits long-term potentiation, and promotes synapse loss. Notably, microglial mitochondrial dysfunction has been shown to impair their capacity to clear A β and to prune aberrant synapses appropriately, further aggravating synaptic pathology. Conversely, restoring microglial mitochondrial function—for example, by enhancing mitophagy or reducing oxidative stress—can shift microglia toward a neuroprotective state and rescue synaptic deficits in AD models.

Astrocytic mitochondria and the metabolic support of synapses. Astrocytes provide critical metabolic and homeostatic support to neurons, including lactate shuttling, glutamate clearance, and ion buffering—processes that all rely on a healthy mitochondrial network. In AD, astrocytic mitochondria show structural abnormalities and reduced respiratory capacity, leading to impaired glutamate uptake and elevated extracellular glutamate, which promotes excitotoxic damage at synapses. Astrocytic mitochondrial dysfunction also compromises the ability of these cells to supply energy substrates and antioxidant precursors (e.g., glutathione) to neurons, leaving synapses more vulnerable to oxidative and metabolic stress. Moreover, astrocytic endfeet are integral components of the neurovascular unit, and mitochondrial failure in these cells can impair blood-brain barrier integrity and neurovascular coupling, thereby reducing nutrient and oxygen delivery to active synapses.

Intercellular mitochondrial transfer: a protective and pathological axis. One of the most striking recent discoveries is that mitochondria can be transferred between cells in the central nervous system. Astrocytes have been shown to release functional mitochondria—either as free organelles or within extracellular vesicles—that can be taken up by neurons. This transfer constitutes a fundamental neuroprotective mechanism: in models of cerebral ischemia and glutamate excitotoxicity, neuron-derived stress signals trigger astrocytic release of healthy mitochondria, which neurons internalize to restore their own mitochondrial pool and support cell survival. Conversely, in some pathological contexts, damaged mitochondria from neurons can be transferred back to astrocytes for degradation (a process termed “mitophagy”), implicating astrocytes as systemic managers of mitochondrial quality control.

In AD, this intercellular mitochondrial transfer appears to be dysregulated. Evidence suggests that A β and tau pathology impair the release of neuroprotective mitochondria from astrocytes while simultaneously promoting the transfer of dysfunctional mitochondria from damaged neurons to microglia, triggering inflammatory cascades. Therapeutic strategies aimed at boosting the astrocyte-to-neuron transfer of healthy mitochondria or at facilitating the clearance of damaged neuronal mitochondria by glial cells are therefore emerging as novel approaches to preserve synaptic integrity.

Implications for the translational framework. Taken together, these findings mandate that any comprehensive translational framework for synaptic mitochondrial health must incorporate the contributions of glial mitochondria. Rational combination therapies of the future should consider not only neuron-intrinsic mitochondrial targets but also agents that restore astrocytic metabolic support, normalize microglial mitochondrial function, and enhance beneficial intercellular mitochondrial transfer within the neurovascular unit. Incorporating these components into the biomarker discovery and patient stratification strategies discussed above will be essential for advancing toward effective, multi-cellular neuroprotective therapies.

**FIGURE 4:**
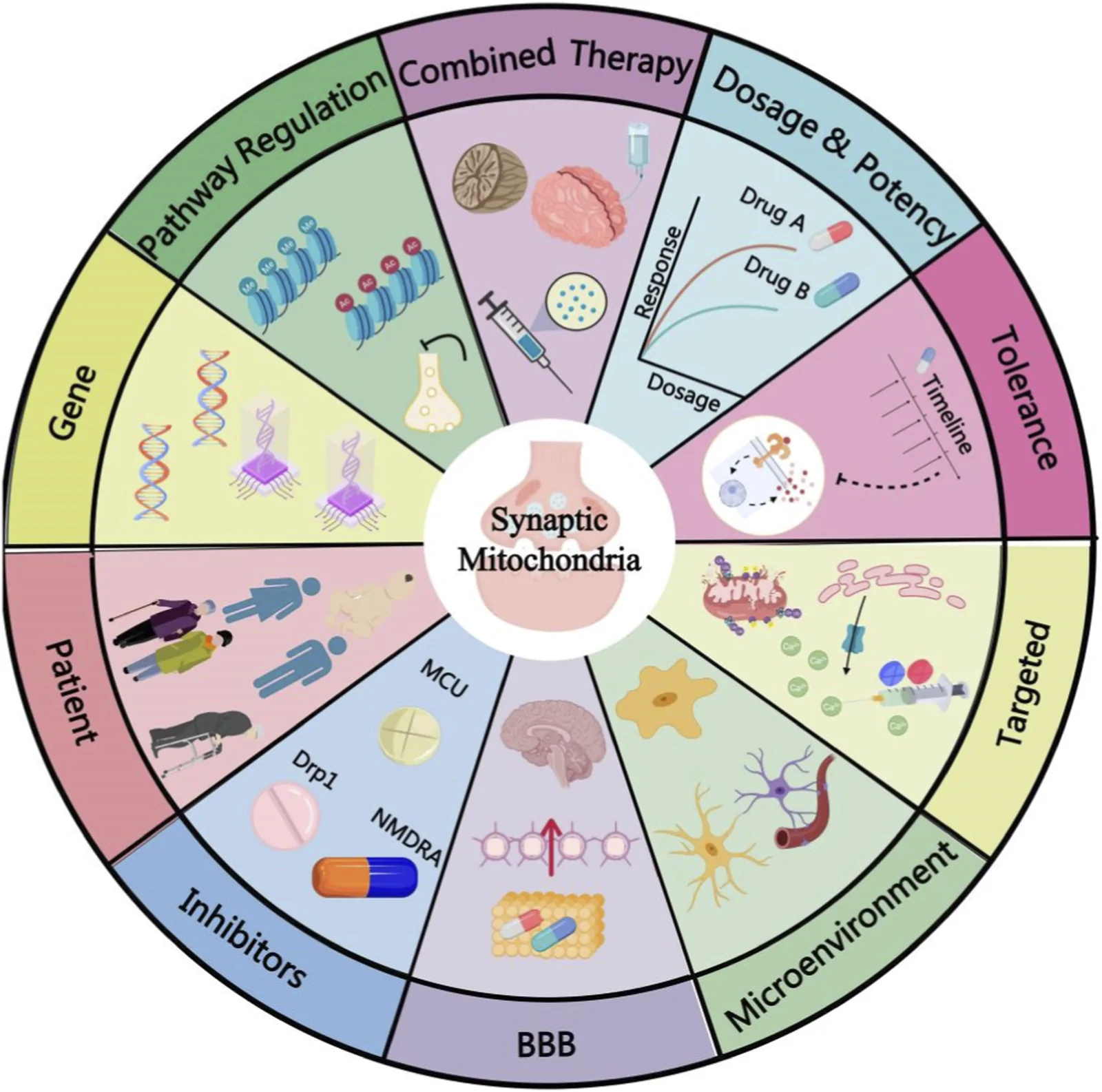
Future directions for synaptic mitochondria-targeted therapies in AD. The left panel highlights basic research priorities: biomarker identification, spatiotemporal dynamics of mitochondrial dysfunction across disease stages, and improved preclinical models. The middle panel outlines therapeutic strategies—antioxidants, metabolic modulators, calcium inhibitors, dynamics regulators, and mitophagy inducers—alongside delivery challenges (BBB penetration and synaptic targeting). The right panel depicts clinical translation: biomarker-guided patient stratification, rational combination therapies, and stage-adapted intervention timing. The bottom panel incorporates emerging multi-cellular perspectives, including glial mitochondrial contributions and intercellular mitochondrial transfer. This integrated framework provides a roadmap from mechanistic discovery to clinical application for next-generation AD therapies.

A central challenge in translating these ideas into practice remains the need to employ high-resolution dynamic imaging alongside multi-omics approaches to characterize spatiotemporal interactions between mitochondrial dysfunction and AD pathology across disease stages in relevant *in vitro* and *in vivo* models and at the single-synapse level (Figure 4). Only through such integrative efforts can we fully unravel the multi-cellular mitochondrial networks that determine synaptic fate in Alzheimer’s disease.

Translation challenges include developing a delivery system targeted at synaptic mitochondria and finding an optimal time window. Identifying biomarkers that reflect synaptic mitochondrial health in the brain will also be crucial to bridge basic research and clinical trials. The scope of research should also include neurovascular units to study how mitochondrial metabolism in non-neuronal cells remotely influences synaptic microenvironment homeostasis via immune and metabolic coupling, revealing targets beyond neurons. Finally, the most useful combination therapy will be synergistic combination therapy, which integrates neuroprotective agents directed at synaptic mitochondria with existing approaches to clear A/tau or control neuroimmunity to create a multi-level, multi-target network to arrest disease progression.

Finally, the synaptic mitochondrial hypothesis provides a framework for understanding the complexity of Alzheimer’s disease, which is not just a pathological model but also a strategic model for future therapeutic development. By deepening mechanistic understanding, overcoming technological kinematics, and implementing synergistic multitarget therapies, interventions focusing on this energy center should provide a practical and promising route to fundamentally change Alzheimer’s disease.
